# Dietary cholesterol supplementation and inhibitory factor 1 serum levels in two dizygotic Smith-Lemli-Opitz syndrome twins: a case report

**DOI:** 10.1186/s13052-020-00924-2

**Published:** 2020-10-28

**Authors:** Maurizio Delvecchio, Biagio Rapone, Simonetta Simonetti, Simona Fecarotta, Graziana De Carlo, Elvira Favoino, Maria Teresa Loverro, Anna Maria Isdraele Romano, Federica Taurino, Edoardo Di Naro, Antonio Gnoni

**Affiliations:** 1 Department of Metabolic Diseases, Clinical Genetics and Diabetology, Giovanni XXIII Children’s Hospital, Bari, Italy; 2 Department of Basic Medical Sciences, Neurosciences and Sense Organs, University of Bari “Aldo Moro”, Bari, Italy; 3 Regional Centre for Neonatal Screening, Children Hospital “Giovanni XXIII”, Bari, Italy; 4 Department of Translational Medical Sciences, Federico II University, Naples, Italy; 5 Department of Biomedical Sciences and Human Oncology, University of Bari “Aldo Moro”, Bari, Italy; 6 Department of Interdisciplinary Medicine, School of Medicine, University of Bari “Aldo Moro”, Bari, Italy

**Keywords:** Smith-Lemli-Opitz syndrome, Inhibitory factor 1, Cholesterol, Lipids, Case report

## Abstract

**Background:**

Smith-Lemli-Opitz syndrome (SLOS) is a rare genetic neurodevelopmental disorder caused by the defect in the 7-dehydrocholesterol reductase. This defect leads to the deficiency of cholesterol biosynthesis with accumulation of 7-dehydrocholesterol. Inhibitory factor 1 (IF_1_) is a well-known mitochondrial protein. Recently, it has been discovered in the human serum where it is reported to be involved in the HDL-cholesterol intake. Here we report the IF_1_ presence in the serum of two paediatric SLOS dizygotic twins treated with dietary cholesterol supplementation.

**Case presentation:**

The patients showed a typical phenotype. They started dietary supplementation with cholesterol when 2 months old. The cholesterol intake was periodically titrated on the basis of weight increase and the twin 1 required a larger supplementation than the twin 2 during the follow-up. When 6.4-year-old, they underwent IF_1_ assay that was 7-fold increased in twin 2 compared to twin 1 (93.0 pg/ml vs 13.0 pg/ml, respectively).

**Conclusions:**

We report, for the first time, the presence of circulating IF_1_ in the serum of SLOS patients, showing different levels among them. Our findings confirm that IF_1_ could be a novel research target in cholesterol-related disorders and also in SLOS, and could contribute to the general debate on IF_1_ as a new modulator of cholesterol levels.

## Background

Smith-Lemli-Opitz syndrome (SLOS) is an autosomal recessive congenital syndrome caused by an inborn error of cholesterol biosynthesis. Cholesterol levels are usually low in SLOS patients, but they can also be normal [1]. More than 150 pathogenic mutations of the 7-dehydrocholesterol (7-DHC) reductase (*DHCR7*) gene have been reported [2–4]. The phenotype may vary broadly, spanning from mild to severe spectrum [5, 6]. SLOS phenotype is not fully characterized and the pathogenetic mechanisms are not completely understood.

The developmental malformations in SLOS may be due to the loss of function of hedgehog proteins, as cholesterol interacts also with them [7]. In addition to cholesterol depletion, the 7-DHC reductase dysfunction leads to the accumulation of the highly reactive precursor 7-DHC [8–10]. To date, there is no proven and effective drug for SLOS patients and dietetic cholesterol supplementation is the only therapeutical approach [11–16].

Inhibitory factor 1 (IF_1_) is a well-known endogenous mitochondrial protein. It works as a regulatory protein, inhibiting the ATP hydrolase activity of mitochondrial ATP synthase [17]. Over the last years, IF_1_ was reported to be present on the external plasma membrane side of many cell lines [18], playing multiple roles. Among them, it has been reported that extracellular IF_1_ blocks the HDL-cholesterol endocytosis by inhibiting the ATP hydrolase activity of plasma membrane-ATP synthase [19]. Recent evidences show that IF_1_ is detectable in the human systemic circulation in physiological conditions showing a positive correlation with serum HDL-cholesterol levels and a negative one with triglycerides [20]. Interestingly, the same research group reported that serum IF_1_ levels, but not HDL-cholesterol, were independently and negatively associated with mortality in long-term male patients with coronary artery disease (CAD) [21]. These findings indicate that IF_1_ might play a role in patients with disorders of cholesterol metabolism.

This case study aims to examine, in the light of the above-mentioned relationship between cholesterol metabolism and IF_1_, its presence in the serum of two SLOS twins in childhood and to ascertain a possible relationship with the difference in the dietary cholesterol requirement. We describe the medical history, discuss the possible role of this protein in the cholesterol metabolism on the basis of previous data from literature, and debate the possible explanations and clinical relevance.

## Case presentation

The two female dizygotic twins were born from caesarean section after 36 weeks of pregnancy obtained by in vitro fertilization and complicated with preeclampsia. Fetal ultrasound showed a symmetrical mild intrauterine growth retardation with an estimated fetal weight below the 10th percentile since the sonographic evaluation performed at 32 weeks of pregnancy. The second and third-trimester scan diagnosed a microcephaly in both fetuses with a head to abdominal circumference ratio below the 3rd percentile and an abnormal shaping of the forehead, but no abnormalities of brain sulcations or further signs of neurological malformations. Cleft palate and micrognathia were suspected for both fetuses during the first trimester screening and confirmed by the anomaly scan, but parents declined any further genetic testing throughout the whole pregnancy. The second trimester scan revealed the presence of syndactyly in both fetuses’ toes: the first suspicion was later detailed by a 3D and 4D scan. The same tool was useful to investigate for the presence of any detectable genital malformation, but no abnormality was reported. Because of the multiple malformations, the assessment of the fetal cardiac anatomy was performed by a specialist in fetal echocardiography: the cardiac structures were described normal as the rest of the anatomy of both fetuses.

At birth, all the malformations detected during pregnancy were confirmed, both fetuses presented microcephaly, second and third toe syndactyly, micrognathia and cleft palate, but the neonatal examination revealed a mild genital hypoplasia, suggestive of SLOS but difficult to diagnose prenatally. The 7-DHC was assayed using gas chromatography-mass spectrometry and was detected at very high concentrations (Table 1). Direct Sanger sequencing of the DHCR7 gene showed that both twins were compound heterozygous for the NM_001163817.1(DHCR7): c.452 G > A (p.Trp151Ter) and NM_001163817.1(DHCR7): c.278 C > T (p.Thr93Met) variants [22] that were detected in the father and in the mother, respectively. Both variants are present in the ClinVar database, with the former being reported as pathogenetic (allele frequency 7.759E-04) and the latter as pathogenetic/likely pathogenetic (allele frequency 4.976E-05). They started a dietary supplementation with cholesterol (approximately 100 mg/kg/day, in compliance with literature suggestions) [23–25] and formula milk.

**Table 1:** Clinical features of the Twins. HC: head circumference; 7-DHC: 7-dehydrocholesterol; IF_1_: Inhibitory factor 1; *BMI* body mass index, *EEG* electroencephalogram, *n.v.* normal values

	Twin 1	Twin 2
Clinical features at birth	Weight 2090 g (− 1.3 SDS)	Weight 2285 g (− 0.6 SDS)
	Length 43 cm (− 1.5 SDS)	Length 43 cm (− 1.5 SDS)
	HC 29.2 cm (− 2.4 SDS)	HC 29 cm (− 2.5 SDS)
	**Apgar** score 8/9	**Apgar** score 6/9
7-DHC at birth (n.v. 0.02–0.29 mcg/ml)	35.6	25.5
**GI tract abnormalities**	None	Pyloric stenosis (4.3 mm) (at 50 days of life)
Heart (at birth)	Persistent ductus arteriosus	Patent forame ovale
Brain	Neurodevelopmental delay, hypotonia	
EEG age: 5.7 years	Unusual background activities and frequents slow spikes in centro-temporal regions, with clear activation during sleep and a sub continuous pattern.	
Congenital acral malformations	Congenital hip dysplasia, bilateral feet hexadactyly, syndactyly of toes 2 and 3 and of toes 5 and 6, lower limbs asymmetry (left > right)	Bilateral clubfoot with syndactyly of toes 2 and 3
Hearing function	Left moderate-severe neurosensorial deafness (diagnosed at 10 months of age)	Normal (6.4 years old)
Auxological assessment (age: 6.4 years)	Height − 5.0 SDS	Height − 3.3 SDS
	Weight − 5.3 SDS	Weight − 3.1 SDS
	BMI −1.99 SDS	BMI −1.51 SDS
IF_1_ levels (age: 6.4 years)	13.0 ± 0.8 pg/ml	93.0 ± 1.1 pg/ml
Cholesterol supplementation age: 6.4 years	77 mg/kg/day	55 mg/kg/day

Total cholesterol was 40 mg/dl (normal value 59–216 mg/dl), 7-DHC 11.66 mg/dl (total cholesterol / 7-DHC ratio 3.26), and 8-dehydrocholesterol (8-DHC) 10.55 mg/dl In Twin 1 before starting the dietary supplementation. The clinical severity score was 35 (classified as typical SLOS), and both the total cholesterol and the total cholesterol / 7-DHC ratio were in the range of typical SLOS [12, 26]. At 3 months she presented hip dislocation and underwent surgery. When 18 months old, tenotomy of left abductors was performed and plaster feet were applied because of bilateral congenital clubfoot. As she presented sleep-wake disorder and developmental delay, electroencephalogram (EEG) was run at the age of 5.7 years. Unusual background activities and frequent slow spikes in centro-temporal area, with clear activation during sleep and a sub-continuous pattern were found. No seizures occurred. She started physiokinesitherapy. Auditory brainstem response showed left mild-severe neurosensorial hypoacusia, stable at 6 years of age.

In Twin 2, total cholesterol before starting the dietary supplementation was 45 mg/dl (normal value 59–216 mg/dl; 7-DHC and 8-DHC not available). The clinical severity score was 40 (typical SLOS), and the total cholesterol level in line with this classification. She presented persistent vomiting. At 50 days of life pyloric stenosis was diagnosed and she underwent surgery. Plaster feet were applied due to bilateral congenital clubfoot. She acquired the sitting position when 2 years old and started to walk and to speak at the age of 3 years. Because of sleep-wake disorder and neurodevelopmental delay, EEG was run at 5.7 years and showed unusual background activities and frequent slow spikes in centro-temporal area, with clear activation during sleep and a sub-continuous pattern. Auditory brainstem response was normal. The clinical findings of the twins are summarized in Table 1.

**Fig. 1:**
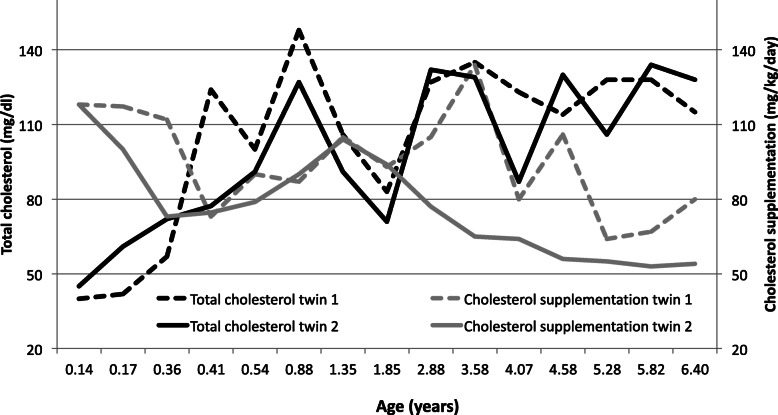
The figure displays the trend of serum total cholesterol (mg/dl) and the cholesterol supplementation (mg/kg/day) in the Twins from the beginning of the treatment until the age of 6.4 years. The aim of the therapy is to keep serum total cholesterol above 100 mg/dl

The twin 1 had a higher level of 7-DHC at diagnosis and a larger requirement of cholesterol intake than the Twin 2. During the follow-up, the cholesterol intake was periodically titrated on the basis of weight and biochemical findings to reach serum total cholesterol of least 100 mg/dl. The dose was about 100 mg/kg/day in both of them at the beginning of the treatment and decreased during the follow-up. When IF_1_ was assayed, it was 80 and 54 mg/kg/day in the twin 1 and 2 respectively. The levels of serum cholesterol and the cholesterol supplementation requirement are displayed in Fig. 1.

At the age of 6.4 years old they underwent two separate blood samplings and remnant sera were aliquotated and used for IF_1_ assay. IF_1_ levels were measured using a sandwich assay with the Human ATPase Inhibitor Mitochondrial ELISA kit (Biovendor, Brno, Czech Republic) following the manufacturer’s instructions. Briefly IF_1_ concentrations (pg/ml) in sera were determined on the calibration curve generated by incubating wells with known concentrations of standard, up to a maximum of 400 pg/ml, and corrected for the dilution factor. First, we measured the serum IF_1_ levels in a control group of twenty age and sex-matched subjects, obtaining a mean value of 70.1 ± 9.5 pg/ml in agreement with the values reported by the manufacturer. Then the analysis of serum IF_1_ level in the twins revealed a 7-fold increase in Twin 2 compared to Twin 1 (13.0 pg/ml in Twin 1 and 93.0 pg/ml in Twin 2) (Table 1). All the serum samples were tested in triplicate.

## Discussion and conclusions

IF_1_ is canonically known as an endogenous mitochondrial protein of 81 amino acids. It works as a regulatory protein, inhibiting the ATP hydrolase activity of mitochondrial ATP synthase [17]. Mitochondrial dysregulation is reported in several pathophysiological conditions especially involving brain pathology [27, 28].

In the last years, activation and/or deactivation of mitochondrial IF_1_ were reported to play a key role in an increasing number of diseases such as cancer, diabetes, and hypo−/anoxia [28]. The ectopic localization of IF_1_ on the outer-face plasma membranes of many cell lines has been highlighted, although the physiology and the clinical meaning are not uniquely defined [18]. Cavelier et al. [19] highlight the role of ecto-IF_1_ in foam cells in the regulation of the reverse cholesterol transport enhancement and/or in the HDL-cholesterol accumulation. The exogenous recombinant IF_1_ seems to inhibit the ATPase activity of plasma membrane ecto-ATPase, reducing the HDL-cholesterol endocytosis in HepG2 cells and rat perfused livers. In the same study, the inhibition of extracellular ATP synthase by IF_1_ increased the circulating HDL-cholesterol [19]. Moreover, the IF_1_ presence in the human serum was unambiguously demonstrated in a cohort of 100 male subjects aged 45–75 years and the reported correlation with the HDL-cholesterol levels was significant although the correlation coefficient was quite low (i.e. *r* = 0.259, *p* = 0.009) [20**]**. IF_1_ serum levels, in male patients with coronary artery disease, were linked with mortality, suggesting that IF_1_ could be a novel target not belonging to classical cardiovascular risk factors and/or variables [21].

The previously reported method of serum IF_1_ analysis had some limitations [20, 21]. The most important is the use of a homemade ELISA assay, based on a polyclonal, self-product antibody, biasing the standardization of the procedure and the possibility of widespread use. The kit used in our study is accurately standardized and validated by the manufacturer in 155 unselected adult donors (e.g. 89 men and 66 women, 21–65 years old). The IF_1_ limit of detection in our study is nearly two-order of magnitude lower than previously published (i.e. pg/ml in our study vs μg/ml as reported by Genoux et al. [20, 21]. This relevant finding should be taken into account, when the relationship of IF_1_ with other clinical and/or laboratory markers is considered (e.g. the previously reported correlation with HDL-cholesterol and triglycerides) [20, 21].

Cholesterol levels in these SLOS twins were very low at diagnosis. Cholesterol plays a key-role in embryo and foetus development and the understanding of SLOS pathogenic mechanisms is still incomplete. Multiple factors are likely to modulate its severity and the result is that a genotype-phenotype correlation is not clearly predictable. Even if the dietary treatment may normalize cholesterol levels, prenatal damage cannot be corrected and mild serum 7-DHC may persist even after years of therapy [29].

Interestingly, in these SLOS twins the difference in IF_1_ levels_,_ higher in Twin 1 than in Twin 2, reflects the difference in the cholesterol supplementation. A possible explanation for these results may be that the lower IF_1_ level detected in Twin 1 account for an increase of hepatic HDL uptake in vivo, which in turn decreases the levels and the particle size of HDL-cholesterol, thus reducing its residence time in serum. This could be one of the mechanisms, which could account for the difference in the cholesterol intake required to get comparable cholesterol levels over the follow-up. The therapeutical approach to SLOS is based on the assumption that cholesterol is the missing end-product of the pathway, so its supplementation may inhibit the de novo pathway, reducing the further production and accumulation of 7-DHC and/or of other cholesterol precursors. The cholesterol supplementation shows a wide variability, ranging from minimal to modest clinical efficacy [29]. The liver enzymes were within the normal range over the whole follow-up (data not shown) suggesting that the accumulation of 7- and 8-DHC did not cause liver impairment. In our patients the dietary treatment has been quite effective as it normalized the total cholesterol levels.

The efficacy of cholesterol supplementation is debated. Some authors reported that SLOS patients could benefit from the use of statin as adjunctive therapy to cholesterol supplementation. It should be noted that other molecules like simvastatin, which inhibits HMG-CoA reductase, were tested in patients with severe SLOS and liver impairment [30]. Nonetheless, in consideration of the potential side effects, statins cannot be considered a safe approach in SLOS. The detection of other molecules, which can be a potential target for new drugs, could be important in the development of a new therapeutic strategy in SLOS.

Our study has some limitations typical of case reports on a rare disease which should be considered. Longitudinal IF_1_ assessment might be of interest to gain further insight about serum IF_1_ role in the biochemical spectrum of the disease, shedding light on its fluctuation during the follow-up in relationship with cholesterol levels and dietary treatment. In advance, a standardized and reproducible serum IF_1_ assay has become recently available and therefore no data are available in literature so far. For this reason, we could not assay serum IF_1_ at diagnosis nor at other stages. On the other hand, the dizygotic twins research design is relevant as it eliminates the environmental factors leaving the genetic contribution to the investigated disorder.

This is the first study that has reported the presence of IF_1_ in the serum of SLOS patients. Our results support the hypothesis that IF_1_ can be a reliable target for further research in SLOS and in general in other pathologies related to an impaired cholesterol regulation. Nevertheless, this is a recent field of research and thus this paper may be relevant for future studies and for those with an interest in cholesterol metabolism and its related disorders.

## Data Availability

Not applicable.

## Abbreviations

SLOS: Smith-Lemli-Opitz syndrome
*DHCR7*: 7-dehydrocholesterol reductase
IF_1_: Inhibitory factor
EEG: Electroencephalogram
